# Inner Branch Endografts in Complex AAA: Case Reports Comparing Off‐The‐Shelf and Custom‐Made Options

**DOI:** 10.1155/cris/8885526

**Published:** 2025-12-23

**Authors:** Pere Altés, Ivan Sánchez, Lucía Martínez, Candela Otero, Carlos Esteban, S. Llagostera

**Affiliations:** ^1^ Department of Angiology and Vascular Surgery, Germans Trias i Pujol University Hospital, Catalonia, Badalona, Spain, germanstrias.org

## Abstract

Complex aortic aneurysm repair has witnessed remarkable advancements through endovascular solutions. In current practice, many endovascular repairs of the abdominal and thoracic aorta rely on patient‐specific endografts designed and manufactured on demand, as their anatomical requirements cannot be met by assembling conventional off‐the‐shelf components. However, the E‐nside system is currently the only commercially available thoracoabdominal endograft that incorporates pre‐cannulated inner branches in an off‐the‐shelf design. The objective of this case series is to describe and compare clinical indications, technical features, and intraoperative outcomes of complex abdominal aortic aneurysm (AAA) treated using the inner branched off‐the‐shelf E‐nside and custom‐made E‐xtra Design MultiBranch endografts under real‐life circumstances. Both endografts were safe for treating complex aortic aneurysms. E‐nside was more suitable for emergent cases due to easy availability and quick access. Patients of the custom‐made series had lower aortic coverage, but radiation exposure did not differ.

## 1. Introduction

Over recent years, endovascular strategies have become central to aortic aneurysm management, driving substantial changes in how these patients are treated. This has markedly reduced procedural invasiveness and broadened therapeutic options worldwide [1, 2]. Despite these advances, several challenges persist, including the impact of tortuous anatomy, cumulative radiation exposure, and the nonnegligible rates of morbidity and mortality still associated with complex repairs [3, 4]. A preliminary version of the early clinical experience with inner‐branch technology was previously shared in abstract form [5].

Successful endovascular repair requires adequate proximal and distal landing zones within healthy aorta, and complex aneurysms frequently demand preservation of visceral perfusion. If challenging anatomy compromises the ability of a fenestrated graft to obtain secure wall apposition or dependable cannulation of the visceral vessels, a branched configuration may provide a more suitable solution.

Although both approaches perform well within their established indications, anatomical constraints—such as severe angulation, marked tortuosity, or limited sealing length—may reduce their effectiveness or feasibility. [6–9] Inner‐branch endografts (iBEVAR) emerged as a response to these limitations, offering a design that combines the practical benefits of fenestrations with the directional stability characteristic of outer branches. This configuration offers reduced aortic coverage and improved handling in anatomically complex or kinked segments, potentially expanding applicability in selected scenarios [10–12].

Spinal cord ischemia remains one of the most severe complications of thoracoabdominal aortic repair. Endovascular treatment provides a degree of protection in comparison with open repair, yet the residual risk is strongly associated with the extent of aortic coverage, even when preventive strategies are implemented [8, 13].

Custom‐made devices are rarely an option in emergencies, since their production timeline does not allow immediate treatment. The aim of this study is to present and compare the indications, technical characteristics, and intraoperative results of complex abdominal aortic aneurysm (AAA) repair using the off‐the‐shelf inner branched E‐nside endograft and the custom‐made E‐xtra Design MultiBranch system in our center [11].

## 2. Methodology

We conducted a retrospective, single‐center, consecutive case series including patients treated for complex aortic aneurysms between January 2021 and December 2022. All individuals provided written informed consent for the use of their clinical information for research purposes. Each patient underwent endovascular repair at our institution using an inner‐branch configuration; in selected cases, an additional thoracic endograft was required depending on anatomical extent. Demographic and anatomical variables, procedural details, and perioperative outcomes were collected from electronic medical records. No written consent has been obtained from the patients as there are no patient identifiable data included in this case report/series.

Two different multibranch systems from Artivion, Inc. were used: the off‐the‐shelf E‐nside Thoracoabdominal Multibranch Stent Graft System and the custom‐made E‐xtra Design MultiBranch device. Treatment allocation was determined by a multidisciplinary aortic committee through shared decision‐making, taking into account anatomical suitability, urgency, and patient‐specific considerations.

The E‐nside graft was selected when the patient’s anatomy fell within the predefined inner‐branch configuration and when immediate availability was clinically advantageous. Eligibility criteria included alignment of the celiac trunk, superior mesenteric artery, and both renal arteries within the device’s longitudinal and circumferential branch windows; target‐vessel diameters compatible with bridging stents; adequate proximal and distal sealing zones; and iliofemoral access capable of accommodating a 24‐Fr delivery system. Because upper‐extremity access is required for target‐vessel catheterization, cases with significant aortic‐arch atheroma or mobile plaque were excluded from E‐nside use.

Custom‐made E‐xtra Design MultiBranch endografts were chosen when patient anatomy did not meet the off‐the‐shelf specifications, when more than four branches were required, or in elective settings where the manufacturing lead time—approximately 22 working days—was acceptable.

Percutaneous bilateral common‐femoral access was obtained under ultrasound guidance, and preclosure was performed using the Perclose ProGlide system (Abbott Vascular). Both devices require a 24‐Fr introducer for femoral delivery. Target vessels were catheterized and stented primarily via upper‐extremity access, although femoral approaches were used when feasible. The E‐nside system incorporates four preloaded catheters to facilitate inner‐branch cannulation, whereas the E‐xtra Design MultiBranch offers two to five branches with customizable lengths and configurations; in contrast, the E‐nside graft has a fixed length of 222 mm.

For each procedure, we recorded the type and length of bridging stents used, total aortic coverage, fluoroscopy time, radiation exposure quantified by dose‐area product (Gy·cm^2^), and all intraoperative or perioperative complications. Statistical analysis was performed using the Chi‐square test for categorical variables and Student’s *t*‐test for continuous variables.

## 3. Cases Series and Results

**Figure 1:**
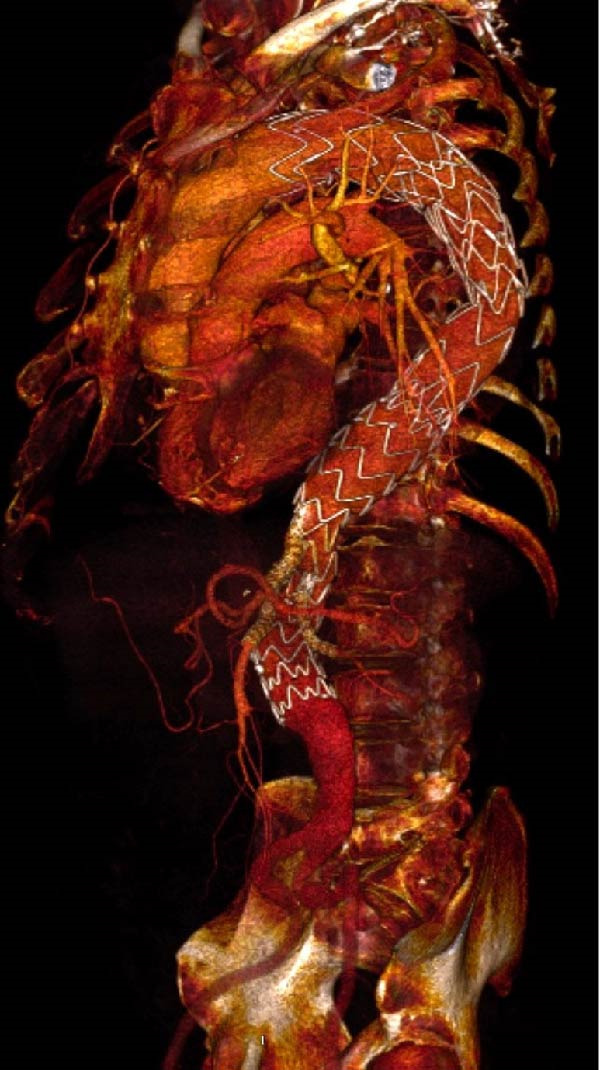
Endovascular repair of a thoracoabdominal aortic aneurysm using the off‐the‐shelf E‐nside Thoracoabdominal Multibranch Stent Graft System.

**Figure 2:**
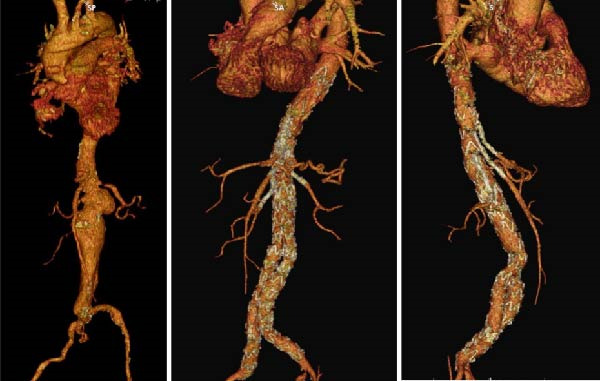
Crawford Type IV thoracoabdominal aneurysm measuring 90 mm treated with a custom‐made E‐xtra Design Multibranch stent graft.

**Figure 3:**
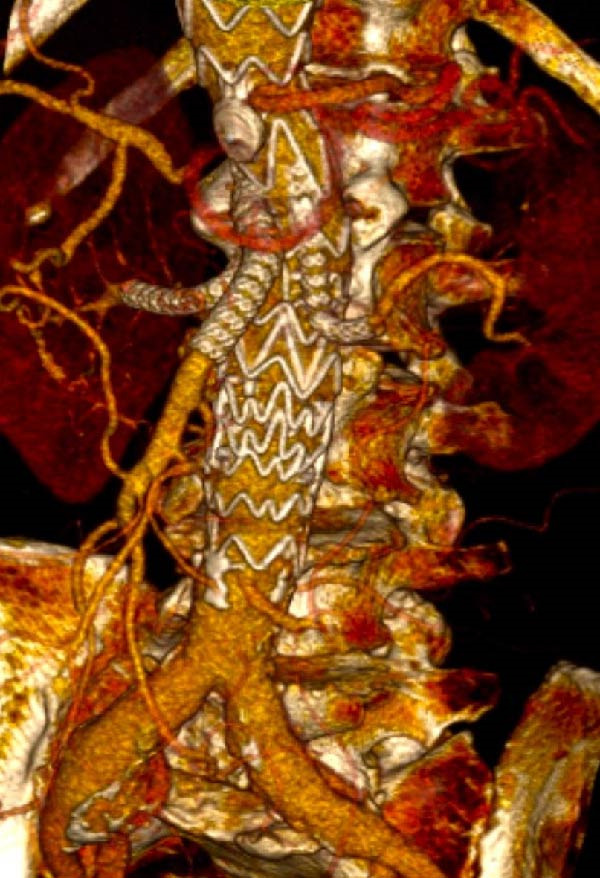
E‐nside thoracoabdominal multibranch stent graft with intentional occlusion of the celiac trunk using an endovascular plug.

We could successfully implant these iBEVAR devices in all the 11 patients (Figures 1 and 2). All patients were male. We detailed the preoperative patient characteristics in Table 1. In the off‐the‐shelf series Case 5, the celiac trunk was impossible to bridge and an Amplatzer plug had to be implanted in the inner branch (Figure 3).

**Table 1:** Baseline characteristics and preoperative diagnoses in the off‐the‐shelf and custom‐made series.

Implanted endograft	Case	Age (years)	Comorbidities	Diagnosis	Maximum diameter (mm)	Ishimura zone of maximum diameter
E‐nside multi‐inner branch	1	73	High blood pressure, diabetes, and dyslipidemia	TAA	65	4
E‐nside multi‐inner branch	2	87	High blood pressure active smoker	TAA	72	9
E‐nside multi‐inner branch	3	78	High blood pressure cerebrovascular infarction and mild cognitive deterioration	TAA	60	5
E‐nside multi‐inner branch	4	70		TAA	78	5
E‐nside multi‐inner branch	5	65	High blood pressure and dyslipidemia	TAA	73	4
E‐nside multi‐inner branch	6	73	High blood pressure, dyslipidemia, and previous treated colonic and prostate cancer	TAA	65	4
E‐xtra Design MultiBranch	1	75	Chronic obstructive pulmonar disease and active smoker	TAA	68	9
E‐xtra Design MultiBranch	2	85	High blood pressure, atrial fibrillation, and ischemic cardiomyopathy	TAA	58	5
E‐xtra Design MultiBranch	3	72	High blood pressure active smoker and chronic kidney disease	TAA	68	5
E‐xtra Design MultiBranch	4	69	High blood pressure, diabetes, atrial fibrillation, and prostate cancer	Type IA endoleak previous EVAR	64	9
E‐xtra Design MultiBranch	5	61	Recent myocardial infarction	Type IA endoleak previous EVAR	97	9

In the off‐the‐shelf series, five over six patients received a thoracic graft prior to the implantation of the inner branched device. Only one patient with a custom‐made device had a thoracic graft implanted before the branched device.

The mean aortic covered length in the off‐the‐shelf series was 393.6 mm, while, in the patients with a custom‐made device, this length was 221.2 mm, and it was significantly lower in these patients (*p* = 0.016). Mean fluoroscopy time was 76 min in both groups. PAD radiation exposure of off‐the‐shelf series was 359 Gy·cm^2^ and custom‐made series was 392 Gy·cm^2^ (*p* = 0.8). We detailed the intraoperative results in Table 2.

**Table 2:** Intraoperative outcomes in the off‐the‐shelf and custom‐made series.

Implanted endograft	Case	Sequential strategy	Previous TEVAR	Aortic coverage (mm)	Fluoroscopy time (min)	Radiation exposure (Gy cm^2^)
E‐nside multi‐inner branch	1	Yes	No	400	55.2	328.77
E‐nside multi‐inner branch	2	Yes	Yes	430	109	378
E‐nside multi‐innerbranch	3	Yes	Yes	300	45	93.4
E‐nside multi‐innerbranch	4	Yes	Yes	280	70	228.3
E‐nside multi‐inner branch	5	Yes	Yes	430	105	886
E‐nside multi‐inner branch	6	Yes	Yes	522	83.5	240.2
E‐xtra Design MultiBranch	1	Yes	No	275	75	570
E‐xtra Design MultiBranch	2	Yes	Yes	363	129	590
E‐xtra Design MultiBranch	3	Yes	No	216	43	284
E‐xtra Design MultiBranch	4	No	No	145	69	268
E‐xtra Design MultiBranch	5	No	No	107	68	250

Regarding the lengths of stents placed in visceral arteries between the series, we observed the following results. For the celiac trunk, patients with both types of endografts required the same mean stent length of 59 mm. In the superior mesenteric artery, the mean stent length was 62.33 mm for the off‐the‐shelf and 55 mm for the custom made series, with no significant difference (*p* = 0.189). In the left renal artery, the mean stent length was 65.5 mm for the off‐the‐shelf and 59.0 mm for the custom made series, showing as well no significant difference (*p* = 0.389). For the right renal artery, the mean stent length was 62.33 mm in the off‐the‐shelf and 51.00 mm in the custom made series, approaching significance (*p* = 0.080).

Regarding complications, the patient Case 4 (off‐the‐shelf series) had a partial spinal cord ischemia that was completely resolved with lumbar drainage of cefaloraquitic liquid. The patient Case 1 of the custom‐made series had a minor stroke with complete recovery. The total number of endografts used were as higher in off‐the‐shelf series (*p* = 0.04).

## 4. Discussion

Inner‐branch configurations offer a practical solution in anatomies that are difficult to treat using standard fenestrated or outer‐branch designs. By incorporating short internal channels that maintain a stable orientation toward the visceral vessels, these systems aim to simplify target‐vessel access and preserve the advantages of both fenestrated and externally branched designs. The combination of this architecture with preloaded catheter systems facilitates reliable visceral cannulation even in anatomically complex or angulated segments. In both the custom‐made E‐xtra Design MultiBranch and the off‐the‐shelf E‐nside configurations, the relatively wide renovisceral body diameter (minimum 24 mm) expands their potential applicability to juxtarenal and pararenal aortic pathology [8, 12–15].

Although our sample size is limited, the present series suggests that custom‐made grafts were associated with shorter total aortic coverage compared with the off‐the‐shelf device. The result is clinically important because longer segments of aortic coverage are strongly associated with a higher incidence of spinal cord ischemia in thoracoabdominal procedures [16–18]. Notably, the only case of spinal cord ischemia in our cohort occurred in a patient treated with the off‐the‐shelf system.

The preloaded configuration of the E‐nside graft requires that all inner branches and target vessels be catheterized before the device can be deployed. This constraint may prolong the visceral reconstruction phase and has theoretical implications for peripheral or spinal ischemia, particularly in patients with limited collateral reserve. Because upper‐extremity access and wire snaring are inherent to the pre‐cannulation strategy, procedural steps may be more complex than with custom‐made alternatives. Nonetheless, in practice, pre‐cannulation often reduces the time required for individual target‐vessel catheterization, and in our cohort, fluoroscopy times and radiation exposure were comparable between groups.

This study has several limitations. It represents a retrospective, single‐center experience with a small number of patients and no control group. Device selection was individualized based on anatomy, urgency, and availability, which introduces potential selection bias. Follow‐up was short and heterogeneous, and outcome data were derived from routine clinical documentation. As a result, any comparison between off‐the‐shelf and custom‐made systems should be interpreted as exploratory.

## Ethics Statement

This study was conducted in accordance with international and institutional ethical standards for retrospective observational research, including the principles of the Declaration of Helsinki (World Medical Association, 2013) and the European Union General Data Protection Regulation (EU 2016/679). The study protocol received favorable approval from the Clinical Research Ethics Committee of Hospital Universitari Germans Trias i Pujol (CEI HUGTiP) during the meeting held on 21 November 2025 (Approval ID: PI‐25−246). The committee accepted the study to be conducted without the need for informed consent, as it meets the criteria for an assistance‐quality study with adequate anonymization and data‐protection safeguards.

## Disclosure

The authors acknowledge that a preliminary version of this work was presented as a conference abstract in the April 2023 Supplement of the Journal of Vascular Surgery. The abstract consisted solely of a brief summary and did not constitute a full publication. The present manuscript provides an expanded and original case report. The previous abstract is appropriately cited in the text [5].

## Conflicts of Interest

Pere Altés discloses a proctoring contract with Artivion, formerly Cryolife/JOTEC, as a potential conflicts of interest. All other authors declare no conflicts of interest.

## Author Contributions

**Pere Altés**: conceptualization, methodology, investigation, data curation, formal analysis, visualization, writing – original draft, writing – review and editing, supervision, project administration. **Ivan Sánchez**: formal analysis, validation writing – review and editing. **Lucía Martínez**: investigation, data curation, writing – review and editing. **Candela Otero**: resources, investigation, witing – review and editing. **Carlos Esteban**: methodology, investigation, resources, writing – review and editing. **S. Llagostera**: supervision, validation, writing – review and editing.

## Funding

No funding was received for this manuscript.

## Data Availability

The data that support the findings of this study are available upon request from the corresponding author. The data are not publicly available due to privacy or ethical restrictions.
